# OLDER ADULTS AND THE WORLD CAFé APPROACH: CROSS-GENERATIONAL INITIATIVES IN RESEARCH AND EDUCATION

**DOI:** 10.1093/geroni/igz038.2239

**Published:** 2019-11-08

**Authors:** Nuelle Novik, Bonnie Jeffery, Tom McIntosh

**Affiliations:** University of Regina, Regina, Saskatchewan, Canada

## Abstract

In Canada, numbers of older adults are considered to be increasing, and by 2036, it is expected that seniors will reach 25% of the total population. Since 2009, the Saskatchewan Population Health and Evaluation Research Unit (SPHERU) has developed an interdisciplinary approach to a community-based research program focused on rural older adults. The world café approach is recognized as collaborative and ideal for encouraging dialogue, sharing knowledge, and developing action plans. Set up like a café, four to six participants at each table engage in a series of three conversational rounds lasting approximately 20 minutes each. At the end of each round, participants move to different tables while the facilitator(s) remain at their original tables. We incorporated a world café approach in three distinct research projects, facilitating a total of five world café events. For each of these events, we also engaged with graduate and undergraduate students who were trained to serve as table facilitators. Participating students represented a variety of disciplines including social work, nursing, and gerontology. Older adults participating in the world café events reported positive experiences and appreciation for the opportunity to discuss new information. Student facilitators identified their participation as a “real life” learning and networking opportunity that enhanced their classroom experiences. Challenges identified included issues related to individual mobility, and issues related to noise and sound quality for those with hearing deficiencies. A community-based approach to research is effective when engaging with this population, and a word café event brings seniors directly into the discussion.

